# Case Report: Infantile Cerebellar-Retinal Degeneration With Compound Heterozygous Variants in *ACO2* Gene—Long-Term Follow-Up of a Sibling

**DOI:** 10.3389/fgene.2022.729980

**Published:** 2022-03-11

**Authors:** Dong Jun Ha, Jisun Park, Go Hun Seo, Kyoungyeul Lee, Young Se Kwon, Ji Eun Lee, Su Jin Kim

**Affiliations:** ^1^ Department of Pediatrics, Inha University Hospital, Inha University College of Medicine, Incheon, South Korea; ^2^ Northwest Gyeonggi Regional Center for Rare Disease, Inha University Hospital, Incheon, Korea; ^3^ 3billion, Inc., Seoul, South Korea

**Keywords:** infantile cerebellar-retinal degeneration, ACO2 gene, aconitase hydratase, optic atrophy, global developmental delay

## Abstract

Infantile cerebellar-retinal degeneration (ICRD) is an extremely rare, infantile-onset neuro-degenerative disease, characterized by autosomal recessive inherited, global developmental delay (GDD), progressive cerebellar and cortical atrophy, and retinal degeneration. In 2012, a biallelic pathogenic variant in *ACO2* gene (NM_001098.3) was found to be causative of this disease. To date, approximately 44 variants displaying various clinical features have been reported. Here, we report a case of two siblings with compound heterozygous variants in the *ACO2* gene. Two siblings without perinatal problems were born to healthy non-consanguineous Korean parents. They showed GDD and seizures since infancy. Their first brain magnetic resonance imaging (MRI), electroencephalography, and metabolic workup revealed no abnormal findings. As they grew, they developed symptoms including ataxia, dysmetria, poor sitting balance, and myopia. Follow-up brain MRI findings revealed atrophy of the cerebellum and optic nerve. Through exome sequencing of both siblings and their parents, we identified the following compound heterozygous variants in the *ACO2*: c.85C > T (p.Arg29Trp) and c.2303C > A (p.Ala768Asp). These two variants were categorized as likely pathogenic based on ACMG/AMP guidelines. In conclusion, this case help to broaden the genetic and clinical spectrum of the *ACO2* variants associated with ICRD. We have also documented the long-term clinical course and serial brain MRI findings for two patients with this extremely rare disease.

## Introduction

Infantile cerebellar-retinal degeneration (ICRD, MIM #614559) is a rare, autosomal recessive, infantile-onset neurodegenerative disease. It is characterized by truncal hypotonia, epilepsy, developmental delay, progressive cerebellar and cortical atrophy, optic nerve atrophy, and retinal degeneration (Spiegel et al., 2012; Sharkia et al., 2019). In 2012, Spiegel et al. analyzed eight patients from two families and reported that homozygous variants of the 1-aminocyclopropane-1-carboxylic acid oxidase two gene (*ACO2*, NM_001098.3) were the cause of ICRD (Spiegel et al., 2012). To date, approximately 44 variants with varying clinical features have been reported. Here, we report a case of ICRD in two siblings cause by compound heterozygous variants in *ACO2*. This report can help expand the genomic and clinical spectrum of *ACO2*-related ICRD.

## Case Description

Two siblings without antenatal and perinatal problems were born to healthy non-consanguineous Korean parents. There was no family history of developmental delay, ataxia, or vision impairment.

### Patient 1 (Older Sister)

**FIGURE 1:**
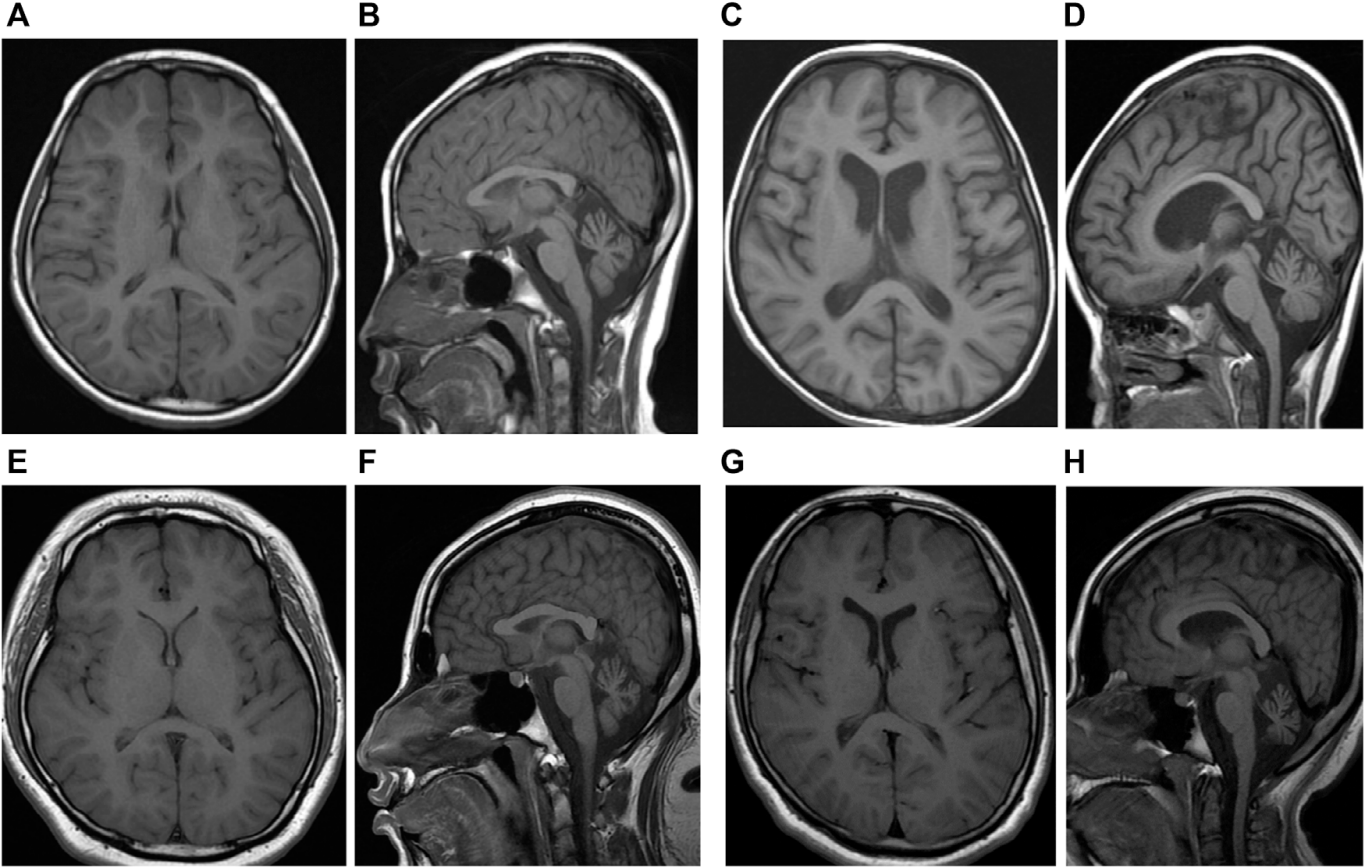
**(A)** T1-weighted axial and **(B)** sagittal image of sibling one performed at 9 years of age show diffuse cerebral, cerebellar and brain stem atrophy and relatively normal ventricle. **(C)**T1-weighted axial and **(D)** sagittal image of sibling one performed at 18 years were not changed significantly. **(E)**T1-weighted axial and **(F)** sagittal image of sibling two performed at 4 years of age show diffuse cerebral, cerebellar and brain stem atrophy and mild hydrocephalus. **(G)**T1-weighted axial and **(H)** sagittal image of sibling two performed at 16 years were not changed significantly.

She was a girl referred at the age of 30 months for occupational and physical therapies for delayed development from early infancy. The Bayley Scales of Infant Development II (BSID-II) test showed global developmental delay (GDD). She had been admitted to the hospital several times for febrile seizures. Her first brain magnetic resonance imaging (MRI) showed no significant abnormalities, and electroencephalography (EEG) suggested partial seizures. She did not take anticonvulsants because she had no partial seizures other than generalized tonic-clonic type convulsion accompanied by high fever. With age, she developed symptoms such as ataxia, dysmetria, poor sitting balance, strabismus, and myopia. Ophthalmic examination revealed atrophy of the bilateral optic nerves at age of 9 years. Follow-up brain MRI showed mild atrophy of the bilateral cerebellum (Figures 1A,B). At 9 years of age, her total intelligence quotient (TIQ), which was evaluating using the Korean Wechsler Intelligence Scale for Children-III was 31, indicating severe intellectual disability. A metabolic work-up, including blood lactic acid, pyruvic acid, amino acids, and urine organic acid tests, showed no abnormal findings. At 19 years of age, she speaks only a few words and can walk or lean against a wall. Further, she has severe vision impairment that allows her to only discern light. Follow-up brain MRI showed no significant changes (Figures 1C,D).

### Patient 2 (Younger Sister)

She also showed GDD with BSID-II at 22 months of age. At 3 years of age, she was admitted to the intensive care unit with a diagnosis of status epilepticus. Her first brain MRI showed no significant abnormal findings, and electroencephalography showed epileptic discharges in the frontal and occipital lobes. She took anticonvulsants, including valproate and topiramate, until 7 years of age. At 4 years of age, she had a decreased response to visual stimuli, and a visual evoked potential study showed optic neuropathy, which led to complete blindness. Similar to her elder sibling, she demonstrated no abnormalities in the metabolic workup. However, her symptoms developed earlier and were more severe than her sister’s symptoms. At 9 years of age, her TIQ was 43, indicating severe intellectual disability. Follow-up brain MRI at 7 years of age revealed hydrocephalus and atrophies of the cerebellum, brain stem, and optic nerve (Figures 1E,F). At 17 years of age, she is unable to walk and cannot speak meaningful words. Follow-up brain MRI showed no significant changes (Figures 1G,H).

### Exome Sequencing

**FIGURE 2:**
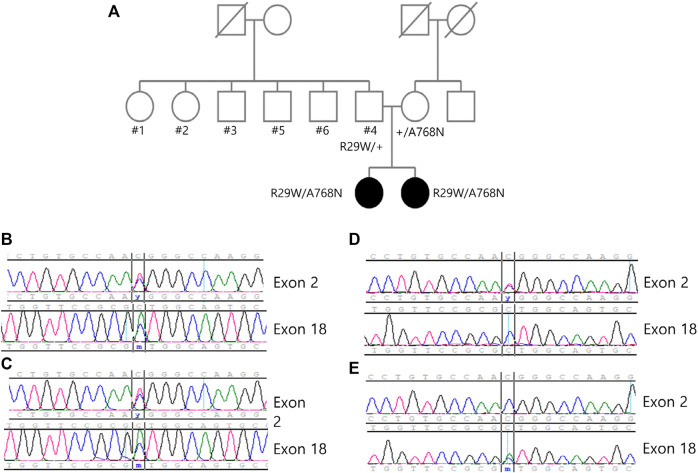
**(A)** Pedigree of the family showing the two affected siblings and unaffected family members. The variants c.85 > T and c.2303C > A can be identified through the sanger sequence of **(B)** sibling 1, **(C)** sibling 2, **(D)** father and **(E)** mother, and each variant is carried from each parent.

We performed exome sequencing (ES) of DNA from the siblings. Genomic DNA was extracted from proband blood. All exon regions of all human genes (∼22,000) were captured by Twist Human Core Exome Kit (Twist Bioscience, South San Francisco, CA, United States). The captured regions of the genome were sequenced using the sequencing ma-chine Novaseq 6,000 (Illumina, San Diego, CA, United States). The raw genome sequencing data analysis, including alignment to the GRCh37/hg19 human reference genome, variant calling, and annotation, was conducted using open-source bioinformatics tools and in-house software. We extracted evidence data on the pathogenicity of variants from previous studies and disease databases, including ClinVar (https://www.ncbi.nlm.nih.gov/clinvar/) and UniProt (https://www.uniprot.org/). The results of ES revealed the following compound heterozygous variants in *ACO2*: c.85C > T (p.Arg29Trp) and c.2303C > A (p.Ala768Asp). These variants were validated by paired-end Sanger sequencing. The variant segregation analysis of the unaffected parents demonstrated that they were heterozygous carriers of each variant (Figure 2). No other homozygous or compound-heterozygous pathogenic or likely pathogenic variants were identified in the known Mendelian disease genes in the exome sequencing data of the siblings.

### Molecular Dynamic Stimulation

**FIGURE 3:**
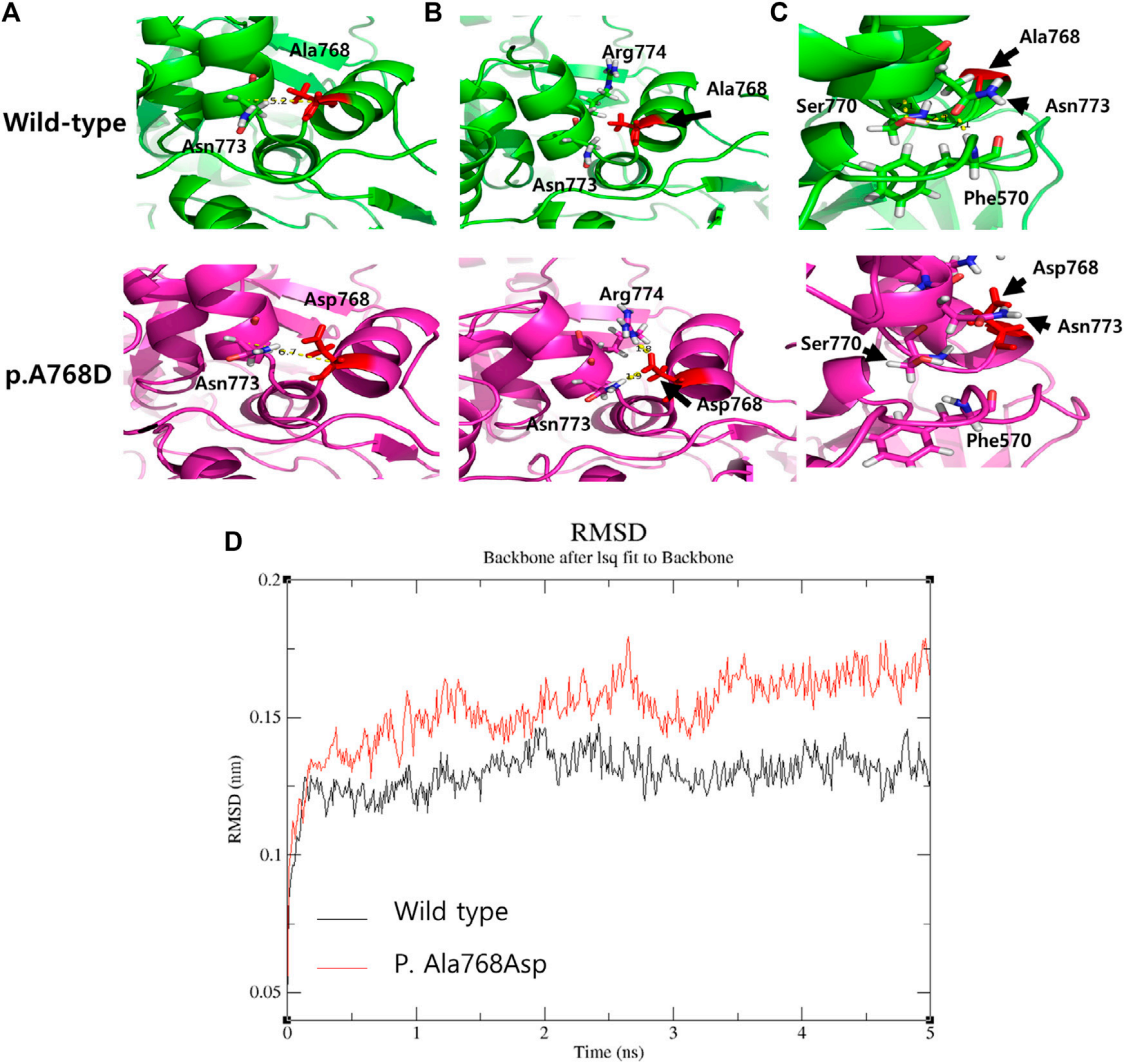
The comparison of The three-dimensional structure between wild type and variant p. Ala768Asp. **(A)** The distance between two helices is predicted to be increased by 1.5 Å (measured by C-alpha distance between two residues). **(B)** Asparagine (773, polar) and Arginine (774, Positive) near the variant site are dragged into Asparagine (768) due to the negative charge of its side chain **(C)** Relocation of Asparagine (773) due to the variant triggers scattering of residues (Ser770 and Phe570) bonded by polar interaction (yellow dotted), thereby destabilizing loop structures. **(D)** The comparison of molecular dynamic (MD) stimulation between wild type and p. Ala768Asp. The Root-mean-square deviation (RMSD) of atomic positions indicated the change in structure over time, and the variant structure (red) shows a large change and unstable pattern compared to the normal structure (black).

Both variants have been reported at an extremely low frequency in large population cohorts (https://gnomad.broadinstitute.org/). The heterozygous variant c.2303C > A on *ACO2* changes the amino acid Ala to Asp at codon 768 in exon 18. However, this has not been reported in large population cohorts (GenomAD). The programs MODELLER (https://salilab.org/modeller/) and GROMACS (https://www.gromacs.org/) were used to visualize and analyze the *ACO2* protein structure. The p. Ala768Asp variant showed probable damage to the protein structure/function (Figure 3A). Structural modeling was performed based on the protein data bank structure (1C96,pdb) of the taurus *ACO2* gene, which has a highly similar sequence (identity = 97%) to the human *ACO2* gene. The distance between two helices is predicted to be increased, and nearby residues (Asp 773, Arg 994) were dragged by charge changes due to the variant, resulting in the relocation of Asp773 and subsequently destabilizing loop structures. Molecular dynamic stimulation was performed to evaluate the effect of this variant on structural stability. The structure of the variant had a larger variation in the root-mean-square deviation compared to the WT (Figure 3B). A missense variant is commonly associated with disease incidence, and the rate of benign missense variants is relatively low. The siblings’ phenotypes were highly specific for this disease. This variant was categorized as likely pathogenic according to the American College of Medical Genetics and Genomics/Association for Molecular Pathology (ACMG/AMP) guidelines (PM2, PP1, PP2, PP3, and PP4) [3]. The heterozygous variant c.85C > T on *ACO2* changes the amino acid Arg to Trp at codon 29 in exon 2. It has been reported at an extremely low frequency in large population cohorts. The allele frequency in GenomAD is 0.00003. *In silico* prediction of this variant showed contradictory results. It is predicted to be disease-causing by Mutation Taster (http://www.muationtaster.org/) and Combined Annotation Dependent Depletion (score: 28.6, https://cadd.gs.washington.edu). Another *in silico* tool, REVEL (https://labworm.com/tool/revel) and MetaSVM (https://sites.google.com/site/jpopgen/dbNSFP) predicted this variant to be tolerated or benign. We could not predict the pathogenicity of this variation using protein structural modeling. However, the heterozygous variant c.85C > T on *ACO2* was co-segregated in affected family members and confirmed as trans with other likely pathogenic variants after the segregation analysis. Further, the phenotypes of the patients were consistent with ICRD. Thus, this variant was categorized as likely pathogenic according to the ACMG/AMP guidelines (PM2, PM3, PP1, PP2, and PP4) (Richards et al., 2015).

## Discussion and Conclusion

In this study, we describe the clinical features of two siblings with two novel *ACO2* variants from infancy to adolescence through a long-term follow-up. *ACO2* (MIM #100850) encodes mitochondrial aconitase hydratase, which converts citrate to isocitrate in the tri-carboxylic acid (TCA) cycle (Mirel et al., 1998; Spiegel et al., 2012). The TCA cycle plays an important role in energy metabolism, and genetic defects in the TCA cycle are associated with various neurodegenerative disorders, including early-onset encephalopathies (Mirel et al., 1998; Briere et al., 2006). The biallelic variant of *ACO2* has rarely been reported and exhibits various clinical manifestations from severe neurodegenerative disorders, such as ICRD, to mild ones, such as isolated optic atrophy 9 (MIM #616289) (Kelman et al., 2018; Sharkia et al., 2019; Blackburn et al., 2020; Gibson et al., 2020). Both of the presented siblings had clinical features commonly seen in ICRD, such as intellectual disability, cerebellar atrophy, and optic nerve atrophy. Although their numbers are limited, patients with ICRD have a wide range of age of onset and severity of phenotype (Sharkia et al., 2019). Table 1 shows the details of our and previously reported ICRD cases. Some reports state that residual aconitase activity in patient tissue or variant-specific assay *in vitro* is associated with clinical severity (Metodiev et al., 2014; Marelli et al., 2018; Blackburn et al., 2020). However, the methods of measuring aconitase activity differ across studies, hindering clinical application of aconitase activity measurement to the diagnosis of *ACO2*-related disorders. There are no useful metabolic biomarkers for the diagnosis of ICRD. Furthermore, sibling 2 demonstrated clinical symptoms, but characteristic radiologic findings, such as cerebellar atrophy on brain MRI, appeared several years later. Therefore, it is not easy for clinicians to suspect this extremely rare disease, ICRD.

**TABLE 1:** A review of the phenotype and genotype of patients with *ACO2* gene variant based on the present studies.

Reference	Our patients	Spiegel et al.	Metodiev et al.	—	—	Sadat et al. (2016)	Abela et al. (2017)	—	Srivastava et al. (2017)	Kelman et al.	Bouwkamp et al. (2018)	Marelli et al.	Sharkia et al.	—	—	—	Fukada et al. (2019)	Ji soo park et al.	Patrick R. et al.
No. of family	1	2	3	—	—	1	2	—	1	1	1	1	5	—	—	—	1	1	1
No. of patients	2	5 + 3	2	2	1	1	2	2	1	2	2	1	1	1	1	2	1	1	2
Onset age	30/22 mon	2–6 months	5/3 years	at birth	5 months	5 months	NA	NA	15 months	2/8 years	3/12 months	NA	7 months	1 day	5 weeks	2/12 months	6 months	2 months	12/20 months
Current age	19/17 years	0.5–18 years	41/36 years	Died at 57/61 days	10 years	3 years	17/14 years	Died at 46 months	18 years	5/9 years	28/14 years	56 years	8 years	Died at 14 years	5 years	8/6 years	Died at 5 years	7 months	12/15 years
Motor skills	Walk with assist	None ∼ sit	Normal	NA	Wheelchair bound	Ataxic gait	NA	NA	Wheelchair bound	Normal	Walk with assist	Walk with assist	Walk with assist	None	None	Walk alone	None ∼ sit with support	None	Impaired fine motor
		With support																	
Cognitive skills	A few words	None ∼ smile	Normal	NA	Smile, recognize family	NA	NA	NA	Full sentence	Normal	A few words	Mild cognitive impairment	A few words	None	None	Some speech	None	None	Dysarthria
Hypotonia (69%)	+	+	−	+	+	+	+	+	+	−	−	−	+	+	+	+	+	+	−
Cerebellar ataxia (80%)	+	+	−	+	+	−	+	+	+	−	−	+	+	+	+	+	+	+	+
Seizures (78%)	+	+	−	+	−	−	+	+	+	−	+/−	−	−	+	+	+	+	+	-
Cortical atrophy (66%)	+	+	−	−	−	−	+	+	−	NA	−	−	−	+	+	−	+	+	+
Cerebellar atrophy (85%)	+	+	−	+	+	−	+	+	+	NA	+	+	−	+	+	+	+	−	+
Optic atrophy (85%)	+	+	+	+/−	+	-	+	+	+	+	−	+	+	+	+	−	−	+	−
Hearing loss (30%)	−	+	−	−	−	+	+	−	−	−	−	NA	−	−	−	−	+	−	−
Ethnicity	Northeast Asian	Arab	French	Algerian	NA	Afro-Caribbean	Arab	Caucasian	Mixed European	NA	Arab	Caucasian	Hispanic/Caucasian	Caucasian	Caucasian	African/Caucasian	Northeast Asian	Northeast Asian	Arab
Genotype	c.2303C > A	c.336C > G	c.220C > G	c.776G > A	c.2208G > C	c.2135C > T	c.336C > G	c.1859G > A	c.2328_2331delGAAG	c.220C > G	c.1240 T > G	c.2135C > T	c.260C > T	c.1181G > A	c.172C > T	c.1787A > G	c.1534G > A	c.1179G > A	c.2050C > T
	c.85C > T		c.1981G > A		c.2328_2331delGAAG	c.1819C > T		c.2048G > T	c.1091 T > C	c.2208 + 1dup		c.940 + 5G > C	c.685–1	c.1722G > A	c.590A > G	c.2050C > T	c.1997G > C	c.1343G > C	c.2153T > C
						—		—				—	_685delinsAA						
Enzyme activity	NA	12%	58/66%	5%	NA	20%	NA	NA	NA	NA	∼20/20%	50%	12%	NA	NA	75/45% of control	15%	NA	NA

D, days; mo, months; wk, weeks; NA, not available; +, present; −, absent.

To date, most of the known *ACO2* variations have been diagnosed based on ES, and most of them are missense variants that require attention for interpretation (Marelli et al., 2018; Sharkia et al., 2019; Blackburn et al., 2020; Park et al., 2020). To interpret a missense variant as pathogenic or likely pathogenic, it is helpful to prove through various web-based *in silico* prediction software applications or protein structure modeling that the variant can induce physicochemical differences or evolutionarily conserved amino acid modification. In this study, both variants in the patients were missense variants, and the heterozygous variant c.2303C > A on *ACO2* was predicted to modify the protein through homology modeling. However, for the other heterozygous variant c.85C > T on *ACO2*, we could not predict pathogenicity using *in silico* prediction tools or protein modeling. For the reasons mentioned above, we could not perform this variant-specific aconitase activity assay. Recently, it was reported that a 12-month-old infant with GDD and epilepsy had compound heterozygous missense variants in *ACO2* through ES, and the c.85C > T on *ACO2* variant was identical to ours (Bruel et al., 2019; Mau-Them et al., 2020). Aconitase activity could not be measured in this case as well. Lack of enzymatic activity measurement is our limitation in proving the pathogenicity of this variant. Nevertheless, the results of the segregation genetic analysis and patients’ clinical manifestations consistent with ICRD offer the possibility that this variant is likely-pathogenic.

It has recently been established that the biallelic pathogenic *ACO2* variant is the genetic cause of ICRD, and although still a rare disease, publications on it are increasing. In patient who shared an identical variant with our case, no ophthalmic problems were found until the age of 2.3 years. In the previous reported literatures, the onset of ophthalmic symptoms varied, and our patient also began to develop ophthalmic problems after the age of 4 years. If *ACO2* variants are found in patients with GDD, regular follow-up for ophthalmic problems is required. In clinical practice, if patients with developmental delay with/without optic nerve atrophy and cerebellar dysfunction, further evaluations such as panel sequencing with *ACO2* genes should be performed. Furthermore, to assist diagnosing patients with mild phenotypes or those who cannot be confirmed through genetic testing, future research is required to determine the association between *ACO2* variants and ICRD and to explore biomarkers that can help obtain the diagnosis.

In conclusion, our cases could help broaden the genetic and clinical spectrum of *ACO2* variants associated with ICRD. In addition, we have shown the long-term clinical course and serial brain MRI findings of two patients with this extremely rare disease.

## Data Availability

The datasets for this article are not publicly available due to concerns regarding participant/patient anonymity. Requests to access the datasets should be directed to the corresponding author.

## References

[B1] Abela L. Spiegel R. Crowther L. M. Klein A. Steindl K. Papuc S. M. (2017). Plasma Metabolomics Reveals a Diagnostic Metabolic Fingerprint for Mitochondrial Aconitase (*ACO2*) Deficiency. PLoS ONE 12, e0176363–15. 10.1371/journal.pone.0176363 28463998 PMC5413020

[B2] Blackburn P. R. Schultz M. J. Lahner C. A. Li D. Bhoj E. Fisher L. J. (2020). Expanding the Clinical and Phenotypic Heterogeneity Associated with Biallelic Variants in ACO2. Ann. Clin. Transl Neurol. 7, 1013–1028. 10.1002/acn3.51074 32519519 PMC7318087

[B3] Bouwkamp C. G. Afawi Z. Fattal-Valevski A. Krabbendam I. E. Rivetti S. Masalha R. (2018). *ACO2* Homozygous Missense Mutation Associated with Complicated Hereditary Spastic Paraplegia. Neurol. Genet. 4, e223. 10.1212/nxg.0000000000000223 29577077 PMC5863690

[B4] Brière J.-J. Favier J. Gimenez-Roqueplo A.-P. Rustin P. (2006). Tricarboxylic Acid Cycle Dysfunction as a Cause of Human Diseases and Tumor Formation. Am. J. Physiology-Cell Physiol. 291, C1114–C1120. 10.1152/ajpcell.00216.2006 16760265

[B5] Bruel A.-L. Nambot S. Nambot S. Quéré V. Vitobello A. Thevenon J. (2019). Increased Diagnostic and New Genes Identification Outcome Using Research Reanalysis of Singleton Exome Sequencing. Eur. J. Hum. Genet. 27, 1519–1531. 10.1038/s41431-019-0442-1 31231135 PMC6777617

[B6] Fukada M. Yamada K. Eda S. Inoue K. Ohba C. Matsumoto N. (2019). Identification of Novel Compound Heterozygous Mutations in *ACO2* in a Patient with Progressive Cerebral and Cerebellar Atrophy. Mol. Genet. Genomic Med. 7, e00698. 10.1002/mgg3.698 31106992 PMC6625133

[B7] Gibson S. Azamian M. S. Lalani S. R. Yen K. G. Sutton V. R. Scott D. A. (2020). Recessive *ACO2* Variants as a Cause of Isolated Ophthalmologic Phenotypes. Am. J. Med. Genet. 182, 1960–1966. 10.1002/ajmg.a.61634 32449285

[B8] Kelman J. C. Kamien B. A. Murray N. C. Goel H. Fraser C. L. Grigg J. R. (2018). A Sibling Study of Isolated Optic Neuropathy Associated with Novel Variants in the *ACO2* Gene. Ophthalmic Genet. 39, 648–651. 10.1080/13816810.2018.1509353 30118607

[B9] Marelli C. Hamel C. Quiles M. Carlander B. Larrieu L. Delettre C. (2018). *ACO2* Mutations: A Novel Phenotype Associating Severe Optic Atrophy and Spastic Paraplegia. Neurol. Genet. 4, e225. 10.1212/nxg.0000000000000225 29564393 PMC5860906

[B10] Metodiev M. D. Gerber S. Hubert L. Delahodde A. Chretien D. Gérard X. (2014). Mutations in the Tricarboxylic Acid Cycle Enzyme, Aconitase 2, Cause Either Isolated or Syndromic Optic Neuropathy with Encephalopathy and Cerebellar Atrophy. J. Med. Genet. 51, 834–838. 10.1136/jmedgenet-2014-102532 25351951

[B11] Mirel D. B. Marder K. Graziano J. Freyer G. Zhao Q. Mayeux R. (1998). Characterization of the Human Mitochondrial Aconitase Gene (*ACO2*). Gene 213, 205–218. 10.1016/s0378-1119(98)00188-7 9630632

[B12] Park J. S. Kim M. J. Kim S. Y. Lim B. C. Kim K. J. Seong M.-W. (2020). Novel Compound Heterozygous *ACO2* Mutations in an Infant with Progressive Encephalopathy: A Newly Identified Neurometabolic Syndrome. Brain Dev. 42, 680–685. 10.1016/j.braindev.2020.07.003 32713659

[B13] Richards S. Aziz N. Bale S. Bick D. Das S. Gastier-Foster J. (2015). Standards and Guidelines for the Interpretation of Sequence Variants: a Joint Consensus Recommendation of the American College of Medical Genetics and Genomics and the Association for Molecular Pathology. Genet. Med. 17, 405–424. 10.1038/gim.2015.30 25741868 PMC4544753

[B14] Sadat R. Barca E. Masand R. Donti T. R. Naini A. De Vivo D. C. (2016). Functional Cellular Analyses Reveal Energy Metabolism Defect and Mitochondrial DNA Depletion in a Case of Mitochondrial Aconitase Deficiency. Mol. Genet. Metab. 118, 28–34. 10.1016/j.ymgme.2016.03.004 26992325 PMC4833660

[B15] Sharkia R. Wierenga K. J. Kessel A. Azem A. Bertini E. Carrozzo R. (2019). Clinical, Radiological, and Genetic Characteristics of 16 Patients with *ACO2* Gene Defects: Delineation of an Emerging Neurometabolic Syndrome. J. Inherit. Metab. Dis. 42, 264–275. 10.1002/jimd.12022 30689204

[B16] Spiegel R. Pines O. Ta-Shma A. Burak E. Shaag A. Halvardson J. (2012). Infantile Cerebellar-Retinal Degeneration Associated with a Mutation in Mitochondrial Aconitase, *ACO2* . Am. J. Hum. Genet. 90, 518–523. 10.1016/j.ajhg.2012.01.009 22405087 PMC3309186

[B17] Srivastava S. Gubbels C. S. Dies K. Fulton A. Yu T. Sahin M. (2017). Increased Survival and Partly Preserved Cognition in a Patient with *ACO2*-Related Disease Secondary to a Novel Variant. J. Child. Neurol. 32, 840–845. 10.1177/0883073817711527 28545339 PMC5515684

[B18] Tran Mau-Them F. Moutton S. Racine C. Vitobello A. Bruel A.-L. Nambot S. (2020). Second-tier Trio Exome Sequencing after Negative Solo Clinical Exome Sequencing: an Efficient Strategy to Increase Diagnostic Yield and Decipher Molecular Bases in Undiagnosed Developmental Disorders. Hum. Genet. 139, 1381–1390. 10.1007/s00439-020-02178-8 32399599

